# A study on the effect of bioactive glass and hydroxyapatite-loaded Xanthan dialdehyde-based composite coatings for potential orthopedic applications

**DOI:** 10.1038/s41598-023-44870-5

**Published:** 2023-10-19

**Authors:** Muhammad Haseeb Nawaz, Aqsa Aizaz, Abdul Qadir Ropari, Huzaifa Shafique, Osama bin Imran, Badar Zaman Minhas, Jawad Manzur, Mohammed S. Alqahtani, Mohamed Abbas, Muhammad Atiq Ur Rehman

**Affiliations:** 1 Department of Materials Science and Engineering, Institute of Space Technology Islamabad, 1, Islamabad Highway, Islamabad, 44000 Pakistan; 2 Electrical Engineering Department, College of Engineering, King Khalid University, 61421 Abha, Saudi Arabia; 3 Centre of Excellence in Biomaterials and Tissue Engineering, Government College University Lahore, Lahore, 54000 Pakistan

**Keywords:** Biochemistry, Biotechnology

## Abstract

The most important challenge faced in designing orthopedic devices is to control the leaching of ions from the substrate material, and to prevent biofilm formation. Accordingly, the surgical grade stainless steel (316L SS) was electrophoretically deposited with functional composition of biopolymers and bioceramics. The composite coating consisted of: Bioglass (BG), hydroxyapatite (HA), and lawsone, that were loaded into a polymeric matrix of Xanthan Dialdehyde/Chondroitin Sulfate (XDA/CS). The parameters and final composition for electrophoretic deposition were optimized through trial-and-error approach. The composite coating exhibited significant adhesion strength of “4B” (ASTM D3359) with the substrate, suitable wettability of contact angle 48°, and an optimum average surface roughness of 0.32 µm. Thus, promoting proliferation and attachment of bone-forming cells, transcription factors, and proteins. Fourier transformed infrared spectroscopic analysis revealed a strong polymeric network formation between XDA and CS. scanning electron microscopy and energy dispersive X-ray spectroscopy analysis displayed a homogenous surface with invariable dispersion of HA and BG particles. The adhesion, hydrant behavior, and topography of said coatings was optimal to design orthopedic implant devices. The said coatings exhibited a clear inhibition zone of 21.65 mm and 21.04 mm with no bacterial growth against *Staphylococcus aureus* (*S. Aureus*) *and Escherichia coli* (*E. Coli*) respectively, confirming the antibacterial potential. Furthermore, the crystals related to calcium (Ca) and HA were seen after 28 days of submersion in simulated body fluid. The corrosion current density, of the above-mentioned coating was minimal as compared to the bare 316L SS substrate. The results infer that XDA/CS/BG/HA/lawsone based composite coating can be a candidate to design coatings for orthopedic implant devices.

## Introduction

Musculoskeletal disorders affect ~ 1.7 billion people globally. The Agency for Healthcare Research and Quality reported 750,000 knee replacements in 2017 in the US^1,2^. Hip and knee replacements are among the most performed surgeries across the globe, with the knee replacement market projected to reach USD 17.45 billion by 2030^2,3^.

Magnesium (Mg), titanium (Ti), cobalt-chromium (Co-Cr), and stainless steel (SS) can be used to fabricate orthopedic implants, including fixators (internal/external), bone plates, prosthesis, etc. Surgical grade SS (316L SS) has properties that make it an attractive option for orthopedic implants. It is bioinert, biocompatible, and has high corrosion resistance, which makes it not produce a strong immunological response or any toxicity in the body. Its mechanical properties (durability and high tensile strength), sterilizability and low cost make it viable for orthopedic implants^4–6^. SS implants are coated with biopolymers and bio-ceramics to promote biocompatibility and bioactivity. Biopolymers such as chitosan (CHI), gelatin, and poly methyl methacrylate can provide a strong network to load nanoparticles like liposomes, graphene oxide (GO), BG, and glycosaminoglycans (GAGs)^7,8^. Through in-vivo and in-vitro testing, biopolymer-based coatings have been proven to increase corrosion resistance and bioactivity. In this context, a robust polymeric network of XDA and CS is proposed to load HA/henna and BG on the facet of stainless-steel implants. XDA possesses an adhesive nature and varying levels of antioxidant activity depending on its environment. XDA also provides more potential crosslinking sites through the presence of aldehyde functional groups, allowing for a strong network for loading nanoparticles^9–11^.

GAGs like heparin sulfate, CS, and hyaluronan were used as biomaterials in different biological applications. CS being the native GAG of bone matrix and cartilage was preferred in the current study. CS possesses anti-apoptotic and anti-inflammatory properties along with boosting production of proteoglycans. Therefore, it is used in various bone diseases to assist tissue regeneration^12–16^. BG are efficient inorganic materials used for bone tissue engineering. They either dissolve or diffuse ions for exchange, promoting osteoblast proliferation, matrix mineralization, and bone marrow stem cell differentiation^17–19^. The release and diffusion of Ca and P ions are vital in prompting growth of apatite-like crystals^20–22^.

Different minerals like octa-calcium, tricalcium phosphate, and HA were used earlier in the related context. HA was used because of its higher bioactivity, stability and osteoinductive property that helps bone regeneration^23,24^. A variety of antimicrobial agents exist in the form of herbal extracts from plants, such as garlic, ginger, clove, thyme, etc., that are used for their antibacterial efficacy^25^. The natural herbs are safer than synthetic drugs to prevent bacterial growth, due to the minimum toxicity of natural herbs. The natural herb used for the reported coating is henna (*Lawsonia inermis).* Henna exhibits a wide range of antimicrobial activities against both Gram-positive and Gram-negative bacteria^26–28^. The core chemical components of henna are 2-hydroxynapthoquinone (lawsone). Quinones are abundantly present in henna and are responsible for their high antibacterial activity^26,29^. EPD was utilized in this study as it is a cost-efficient and effective coating technique, that employs an electric field to move and deposit the charged particles/molecules over the electrode being charged contrarily. Deposition potential along with deposition time directs the structure, topography and width of stated coating^30–32^. EPD can deposit bioceramics (BG, HA, and Ca phosphate cements), biopolymers (XDA, CS, CHI, alginate etc.), and the co-deposition of both from single suspension^30,32–35^. The said polymers or their matrix/network encapsulating drugs/bioceramics can enhance the resistance of 316L SS toward corrosion^36^. Souza et al.^37^ fabricated CHI/Xanthan (Xn)-based scaffolds as periosteum substitutes and it was shown that scaffolds exhibited osteoinductive behavior. Molaei et al.^38^ showed that the CHI/BG/HA/halloysite nanotube composite coatings exhibited *in-vitro* bioactivity upon immersion in SBF^38^. Arias et al.^13^ developed CS-CHI/BG coatings and found them to be highly bioactive, corrosion resistant in addition to the suitable adhesion strength and wettability for orthopedic applications. Rehman et al.^39^ deposited lawsone-loaded BG/CHI on polyetheretherketone/BG layer and reported them to be antibacterial (against *staphylococcus carnasous*), bioactive, and corrosion resistant.

In this study, a novel polymeric complex of XDA/CS was formed, which is expected to mimic extracellular matrix (provide structural support for osteointegration) and can enhance the adhesion with substrate. BG in the composite coatings is expected to induce apatite-like crystals and stem cell differentiation while HA was supposed to enhance osteoinduction rate. Lawsone was introduced in the coating to prevent biofilm formation. Thus, the proposed composite coatings system can induce antibacterial, bioactive, corrosion resistant, and biocompatible properties for orthopedic implant devices. Herein, we used the XDA and CS (polymeric complex) as a matrix to load bioceramics and herbs, which was not used earlier. HA/BG/Henna-loaded XDA/CS coatings were deposited on 316L SS implants. The deposited coatings presented suitable mechanical, electrochemical, and biological properties for the potential coatings to be employed on orthopedic implants.

## Materials and methods

### Materials

Xanthan (Xn), Sodium meta periodate (SMP, > 99%), Chondroitin Sulphate (CS) sodium salt from shark cartilage, sodium metasilicate (Na_2_SiO_3_·9H_2_O, 98% pure), calcium nitrate tetrahydrate (Ca (NO_3_)_2_·4H_2_O, 99% pure), citric acid (C_6_H_8_O_7_, 98% pure), sodium nitrate (NaNO_3_, 99% pure), phosphoric acid (H_3_PO_4_, 99.9% pure), sulphuric acid (H_2_SO_4_, 99.9% pure), Ethylene glycol (EG, > 99% pure), Ethanol (Molecular weight 46.07 g/mol, 99% pure), Acetic Acid (AA, Glacial 100% pure), and Hydroxyapatite (HA) powder were used as received from Sigma-Aldrich Chemie (Sigma Aldrich, Steinheim Germany). Henna was purchased from Saeed Ghani (SG).

### Sample preparation

The 316L SS strips were cut into the rectangular (length—3 cm, width—2 cm, thickness—0.1 cm) and disc shaped (diameter—10 mm, thickness—1 mm) samples using meta cut (METKON302). The disc shape samples were used for antibacterial studies. Afterward, the samples were cleaned and, abrasive silicon carbide papers (having different grit sizes: 120, 240, 360, 400, 800) were used to polish the said samples (120, 240, 360, 400, 800). The bi-distilled water was drawn over the sample`s surface to wash out the impurities/debris. The polished surface of the sample was etched out using 70% H_2_SO_4_ and 30% H_3_PO_4_ for 25 s followed by rinsing in bi-distilled water^40–42^.

### Suspension preparation

#### Synthesis of Xanthan dialdehyde (XDA)

XDA was synthesized by the oxidation of Xanthan Gum (XG) with SMP in a dark controlled environment at 60 °C and stirred for 4—6 h^43^. Guo et al.^43^ and Ngwabebhoh et al.^10^ outlined the mechanism of XDA formation by using 9.6 W/V% (0.096 g/mL) SMP solution. 9.6 W/V% SMP solution concentration for approximately 45% aldehyde content for XDA was reported to have the highest tensile properties. Using ratio stoichiometry, 0.5 W/V% XG required 0.096 g/mL SMP for 45% XDA content (a similar approach was employed in^43^). A portion of 0.125 g of Xn was liquified in distilled water (25 mL) at 60 °C (0.5 W/V% XG). The SMP solution was made using 0.96 g (9.6 W/V%) of SMP in 10 mL of distilled water. Later, XG was oxidized by SMP at the concentration of 0.096 g/mL. The solution was stirred for 4–6 h in a dark environment, following^41^. The reaction was then quenched using 240µL EG, following^10^.

#### Preparation of 45S5 bioglass (BG)

BG of composition: P_2_O_5_ 6%, CaO 24.5%, Na_2_O 24.5%, SiO_2_ 45% (mol%) was synthesized by first dissolving diatom biosilica (prepared following^44^) in 35% C_6_H_8_O_7_ solution. The mixture was magnetically stirred for 2 h under 120 °C. Following the complete dissolution of diatom biosilica, H_3_PO_4_ was added and magnetically stirred for 45 min. Thereafter, NaNO_3_, was added followed by the addition of Ca (NO_3_)_2_·4H_2_O which again was stirred for an hour. The attained gel was treated following the procedure executed in^45^. It was then placed in a muffle furnace (Muffle furnace LMF-E21 manufactured by Labtron) at a constant temperature increment and abatement of 10 °C/min. For 2 h, gel was kept at 700 °C followed by sintering for 3 h at 950 °C. The procedure adopted was similar to Adams et al.^44^.

#### Final preparation of suspension

**Figure 1:**
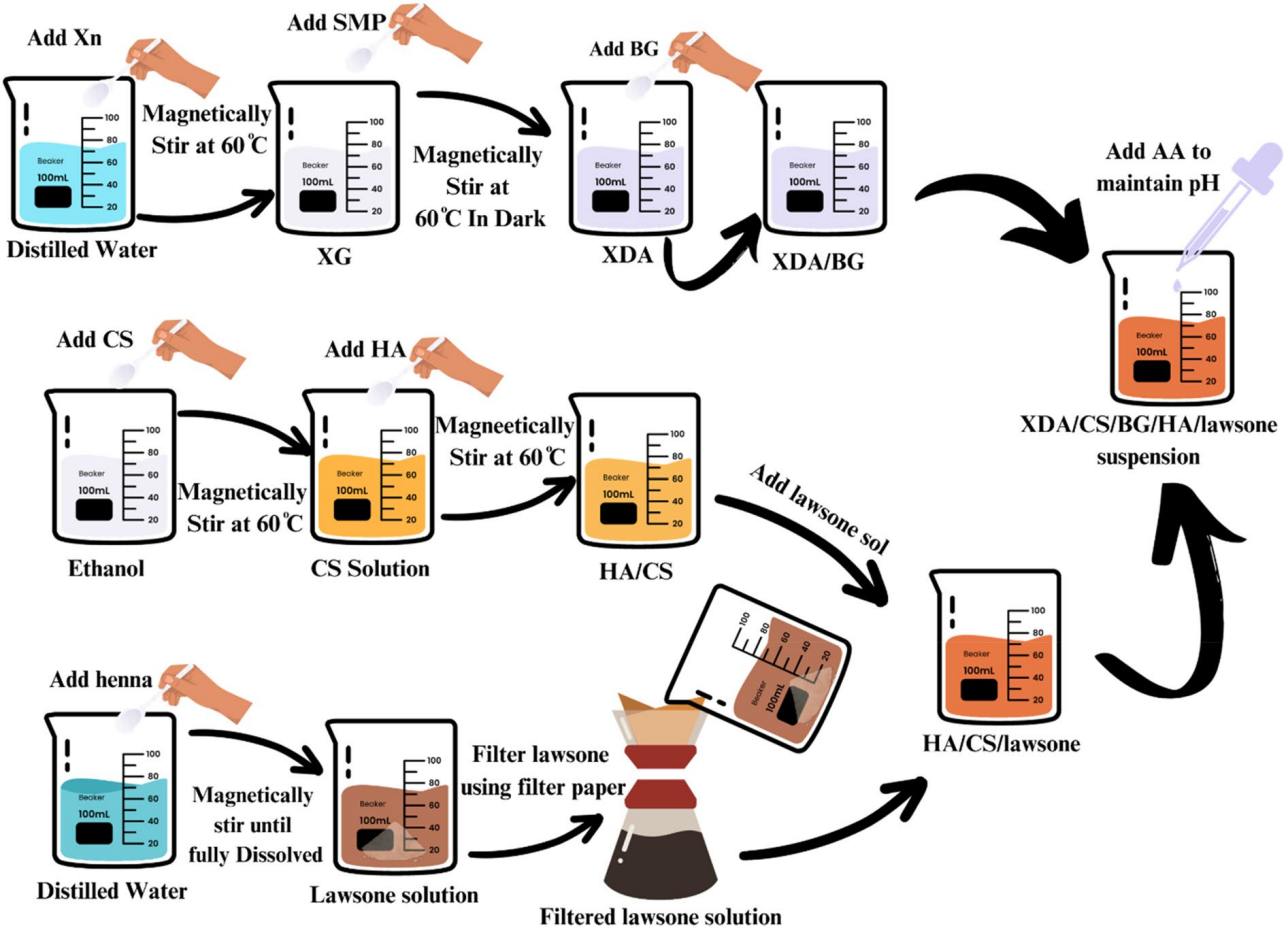
Schematic representation of the XDA/CS/BG/HA/lawsone suspension preparation steps conducted prior to the EPD process.

First, BG (0.125 g) was added in XDA solution (0.5 w/v%) and continuously stirred for 6–7 h at 25 °C to disperse the BG particles. Next, 0.25 g of CS was added to the 25 mL of ethanol (1 w/v%). Later, 0.125 g of HA was added and stirred for 2–3 h at 80–100 °C and sonicated for 2.5 h^46^. In addition, 0.3 g of henna was dissolved in 15 ml of ethanol (2 w/v%)^47^ and the solution was filtered using filter paper. The filtered 10 mL lawsone solution was then added to 25 mL of HA/CS solution (5:2) as shown in Fig. 1, and subsequently stirred for 30 min at 60 °C.

The final suspension for EPD was prepared by using HA/CS/lawsone solution, and XDA/BG solution (an overview is presented in Fig. 1). XDA/BG and HA/CS/lawsone solutions (1:1) were mixed and magnetically stirred at room temperature. The pH of the resulting XDA/CS/BG/HA/lawsone suspension was maintained at ~ 4.5 using AA.

### Electrophoretic deposition

**Figure 2:**
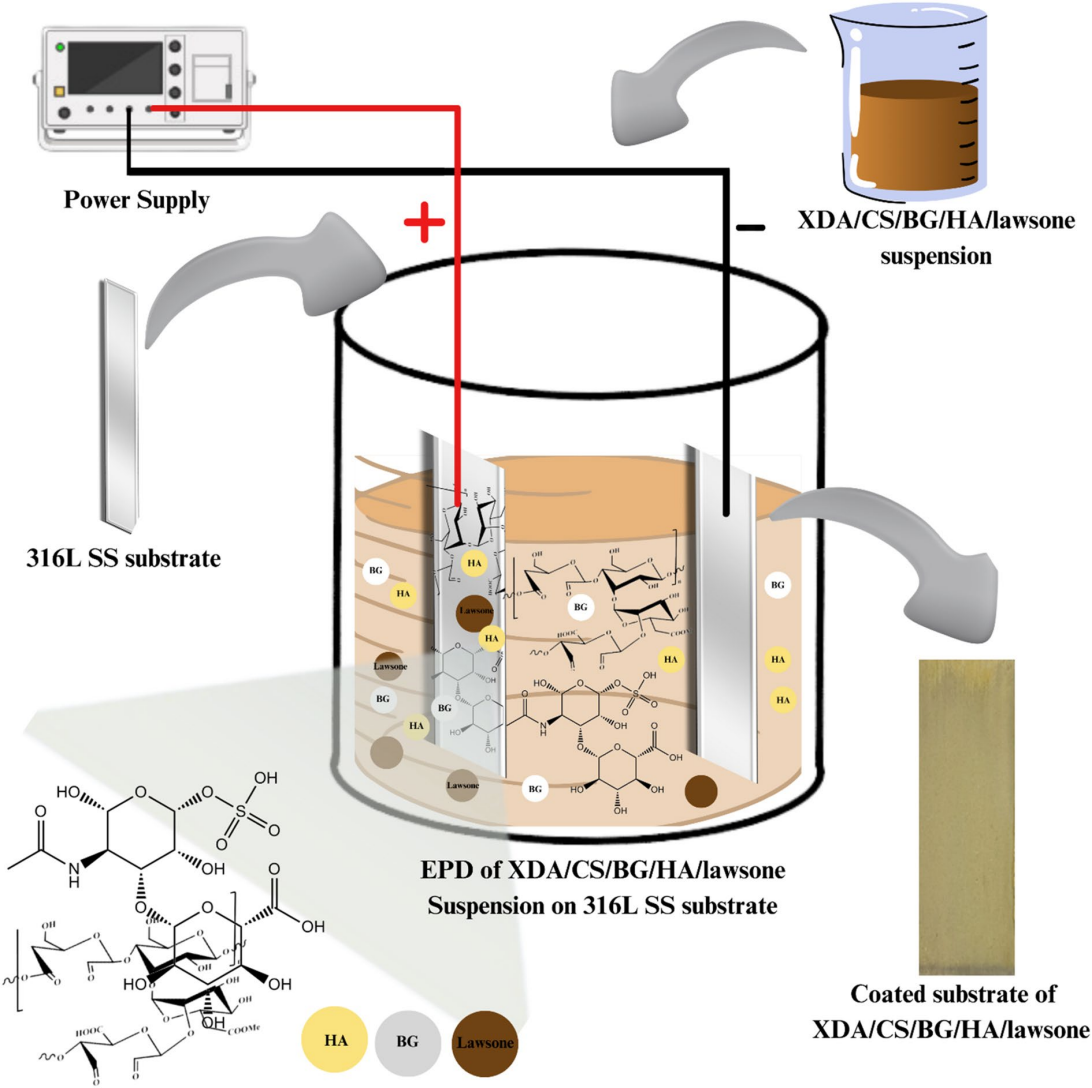
Schematic representation of Direct current EPD setup showing the deposition of XDA/CS/BG/HA/lawsone composite coatings deposited on 316L SS substrate.

A 316L SS was used as substrate (cathode) and counter-electrode (anode). The anode–cathode distance was 10 mm. The coating thickness and the control over surface crack were optimized by tuning the applied deposition voltage and the deposition time given during the EPD process. The voltage was increased in 5 V increments, from 5 to 25 V. A series of experiments were conducted through a trial–error approach (the deposition time and deposition voltage were varied) which yielded the optimal coatings (crack-free and uniform). A continuous voltage of 20 V was applied for 10 min in that particular context as described in Table 1 and Fig. 2. Similar EPD parameters were used in^48,49^ to optimize CHI-based composite coatings.

**Table 1:** EPD Parameters and the suspension composition used for the optimization of EPD process.

Sr no	Suspension (W/V%)					EPD parameters		Results	
	XDA	CS	BG	HA	Lawsone	Deposition time (min)	Voltage (V)	Coated	Adhesion
1	1	1	0.5	0.5	2	5	10	⨂	–
						5	20	⨂	–
						15	15	⨂	–
						15	20	⨂	–
2	0.75	0.75	0.5	0.5	2	5	10	⨂	–
						5	20	✓	⨂
						15	15	⨂	–
						15	20	✓	⨂
3	0.5	0.5	0.5	0.5	2	5	10	⨂	–
						5	20	Thin	✓
						15	15	Thick	⨂
						15	20	Thick	⨂
						10	20	✓	✓

### Ethical approval

This article does not contain any studies with human participants or animals performed by any of the authors.

## Characterization of XDA/CS/BG/HA/lawsone coatings

### Morphological properties

HA/BG/lawsone loaded XDA/CS coatings were investigated optically to check the optimal deposition. By varying time of deposition and voltage applied the variation in levels/thickness of deposition was also investigated. The facet morphology, topography, and thickness of coatings on 316L SS were examined by field emission scanning electron microscopy (FESEM, MIRA, TESCAN). Coated samples of 3 cm × 2 cm × 0.1 cm (rectangular) were examined at various magnifications after being fixed to SEM stubs. To prevent charging effect, the samples were sputtered with Au (Q150/S by Quorum Technologies) before FESEM analysis.

### Adhesion test

A tape test was used to gauge the composite coating's (n = 5) adherence strength to the substrate. According to ISO 2409 and ASTM D3359-17 standards, a crosshatch adhesion test was performed. The CC2000 basic cross hatch cutter was used to create scratches on the coated substrates. The adhesive tape was applied for 90 s on the substrate and removed afterwards maintaining an angle of ~ 180° between the hand and the coated surface. The quality of the adhesive strength was then assessed by comparing the scratches to ASTM Standards^50–52^. The ASTM D3363-20 standard was followed to conduct the pencil scratch hardness test (n = 5), following^53^. Standard holders with grades ranging from 8B (soft) to 2H (hard) pencils were moved over the coated substrate at an angle of 45°. The test continued until the pencil could no longer starch the covered substrate. Following the ASTM B571-97 standard, the adhesiveness of the said coating was analyzed using the bend test (n = 5). Composite coated samples were bent into a 180° U shape using pilers. Composite coatings were evaluated for their adhesive strength by studying their delamination and deformation capacities. A microscope (Novex, Holland) was used to examine the distorted samples after the bending test^54^. Scratch tests were conducted on the composite coating samples with Revetest mode (using a CSM Revetest machine). An indenter with a 200 m diameter was used to apply a progressive load (0.5–4 N) to the coated surface for scratch testing. Indenter speed and scratch length were 5.2 mm/min and 5 mm, respectively. The sample was run on eight separate tracks. By measuring acoustic emission (AE) and through optical observations critical loads were identified. The minimal load at which the initial crack begins to appear was denoted as the critical load (L_C1_) while increase in load lead to the complete delamination and that supremely enhanced load was referred as critical load (L_C2_)^55^.

### Surface properties

#### Surface roughness

The coated XDA/CS/BG/HA/Lawsone and uncoated 316L SS samples were characterized in terms of average surface roughness (Ra), maximum profile peak height (Rt), and average maximum profile (Rz) by employing stylus profilometer (TMR 360). Three measurements were taken for each set of samples and the average value with the standard deviation was reported. The microscopic tip stays in contact with the surface and moves horizontally. However, the tip's vertical movement was recorded and enhanced. It was mounted vertically, using a stylus profilometer. As a result, the stylus is positioned above the part to be measured. On the surface of both samples, the stylus moved 5 mm.

#### Contact angle

Distilled water was used to measure surface wettability. Distilled water droplets of 5 µL were used to quantify the contact angle of the bare 316L SS, XDA coated 316L SS, and XDA/CS/BG/HA/lawsone coated 316L SS substrate. A micropipette was used to drop the droplet on bare 316L SS, XDA coated 316L SS, and XDA/CS/BG/HA/lawsone coated 316L SS. Images were taken after every 5 s for 30 s and analyzed through ImageJ software using drop analysis libraries (LB-ADSA)^56,57^. Tests were repeated five times, and averaged values were reported along with standard deviations.

### Chemical properties

#### Energy dispersive spectroscopy (EDX) analysis

The EDX (OXFORD) installed with FESEM (MIRA, TESCAN) was employed to access the elemental composition of XDA/CS/BG/HA/lawsone coatings (acceleration voltage was kept at 25 kV).

#### FTIR

The functional groups in XDA/CS/BG/HA/lawsone coating were evaluated using OMNIC paradigm software of an attenuated total reflection Fourier transformation infrared (ATR-FTIR) spectroscope (Thermofisher Nicolet Summit Pro). The transmittance spectrum was obtained in the region of 4000–400 cm^−1^ with 4 cm^−1^ resolution after omitting noise.

#### Corrosion behavior

The Gamry instrument (Potentiostat/Galvanostat/ZRA, reference 600) evaluated the corrosion resistance of the composite coatings and bare 316L SS. Gamry device contains three electrodes: working electrodes (coated/uncoated substrates), counter electrode (graphite), and reference electrode (Ag/AgCl). SBF was used as an electrolyte at 37 °C. At 2.5 mV/sec scanning rate, potentiodynamic polarization scans were carried out at ± 500 mV potential. Tafel plot corrosion potential was calculated as (E_corr_) and corrosion current density was calculated as (I_corr_). During an EIS test, an open circuit potential was used with 10 mV perturbation. Frequencies were kept between 100 kHz and 1 MHz. In each case, with measurements in triplicate^58,59^.

### Biological studies

#### In-vitro bioactivity studies

The biocompatibility (generally ability to bind or attach bone cells in case of orthopedic implants) of XDA/CS/BG/HA/lawsone coatings was accessed using SBF synthesized by using the procedure outlined by Kokubo et al.^60^.The composite coatings (1.5 cm × 1.5 cm) were submerged in SBF filled falcon tubes and placed in an shaking incubator (30 rpm) for 1, 7, 14, 21 and 28 days at 37 °C. To simulate physiological conditions, the SBF was replaced every 24 h. After the stated periodic time interval of a week, coated strips were carried out of falcon tubes, rinsed with distilled water and processed for further characterization. SEM and FTIR analyses were conducted on the said samples both before and after immersion in SBF.

#### Antibacterial studies

The basic qualitative antibacterial efficacy of XDA/CS/BG/HA coated 316L SS (Control) and the XDA/CS/BG/HA/lawsone composite coated sample was investigated using the Kirby–Bauer test. Henna was loaded in two different concentrations 0.01 mg/mL and 0.02 mg/mL as of literature and subsequent coatings were tested^61^. Nutrient Agar (Oxoid-UK) was used to prepare agar medium, which was then autoclaved at 121 °C for 20 min, subsequently; 20 mL of prepared media was transferred to the sterile petri dishes. Composite coated and XDA/CS/BG/HA 316L SS were exposed to UV light for an hour. 20 µL of *S. aureus* and *E. coli* owing optical density (OD_600_) of 0.015 ± 0.002 was rolled out over solidified agar medium using a sterilized glass slider. Coatings were then placed over the uniformly distributed bacteria in such a way that coated side-faced bacteria, followed by round the clock incubation at 37 °C. Afterward, Pictures were taken, by using ImageJ software the inhibition zone was measured.

## Results and discussions

### Morphological analysis

**Figure 3:**
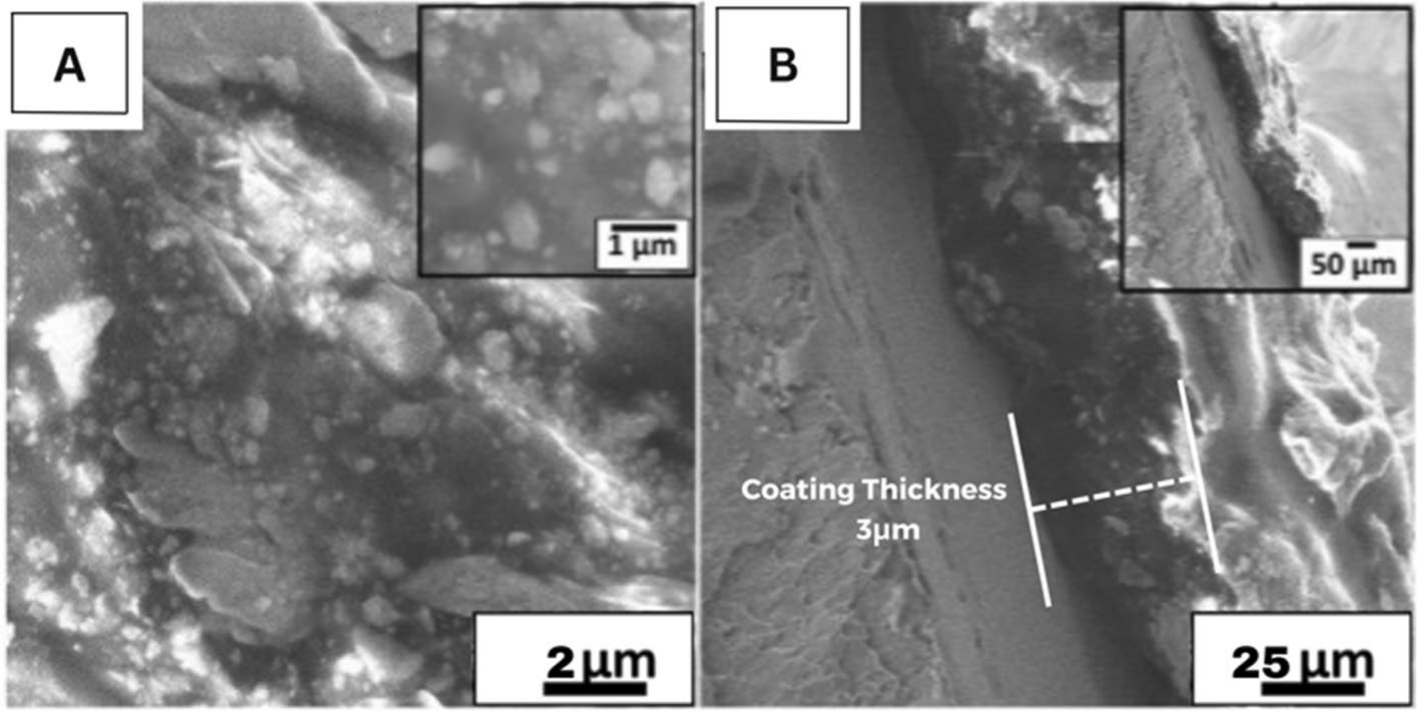
SEM images (**A**) shows uniformly distributed and induced BG and HA particles, respectively at 5 kX and 150 kX magnification, (**B**) shows cross-sectional SEM image of XDA/CS/BG/HA/lawsone.

The surface topography of the XDA/CS/BG/HA/lawsone composite coating deposited on 316L SS was evaluated using FESEM. SEM micrographs of composite coating at low and high magnification revealed a plain, homogeneous, and all-alike coating with no cracks on the surface, as shown in Fig. 3A, B. Through the decomposition of water into hydrogen and oxygen (O) (electrolysis), that usually occurs during EPD, a limited number of micropores created. The coating exhibited uniform distribution of BG and HA particles (FTIR and EDX verified the existence of HA and BG) in the polymeric matrix due to invariable particle size in final suspension^39^. The FESEM images show the agglomeration of BG and HA particles (Fig. 3A). The outcome of the current study is like the one revealed by^62,63^. HA particles are induced and embedded in the polymeric matrix depicting quasi spherical morphologies. The pores present in the polymeric matrix are filled by HA particles and their agglomerates, ultimately intensifying the mechanical stability of the coating. Similar morphologies for HA-based composite coating were proclaimed by Ahmed and Ur Rehman et al.^64^ and Louvier-Hernández et al.^65^. The cross-sectional micrographs of XDA/CS/BG/HA/lawsone composite coating authenticates the uniform coating with thickness of ~ 3 μm, as shown in Fig. 3B. Similar coating thicknesses were reported in previous studies^66,67^.

### Adhesion tests

#### Tape test

**Figure 4:**
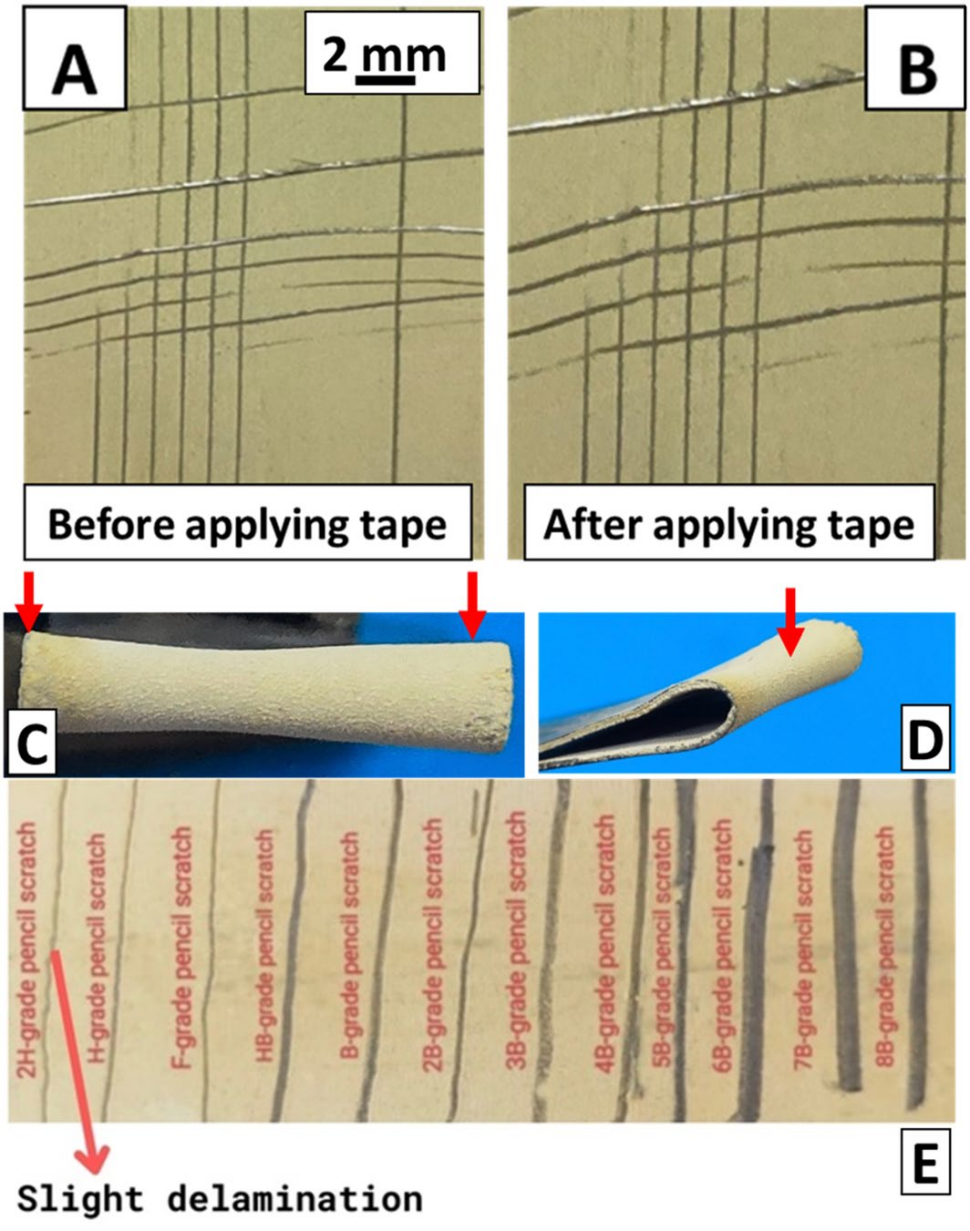
The results of the adhesion strength test: (**A**, **B**) Images of the Tape test before and after Tape test, (**C**, **D**) Images showing the results of the bend test (red arrows represent the area of maximum stress), (**E**) Image showing the result of the pencil hardness test.

Adhesion tests are conducted on composite coating to evaluate their mechanical strength. Samples that underwent tape testing were analyzed using an optical microscope (Novex, Holland) and categorized using a tape test according to ASTM D3359 (Fig. 4). First, we scratched the coated portion of the substrate with a crosshatch cutter, as shown in Fig. 4A. After 90 s, the tape was quickly removed and the image was acquired Fig. 4B. 4B adhesion strength was achieved by the composite coatings (only minor delamination of 5%); this implies that at intersections, small fragments of the coating become dislodged, impacting less than 5% of the surface area, as per the ASTM standards^68^. Prior studies shows that HA/BG-based composite coatings rated as 3B (approximately 5–15% of the lattice area suffer from small flakes detaching along edges and cuts) shows good adhesion strength but in our case coating adhesion rated as 4B much good adhesion strength than already reported studies. The robust adhesion strength observed in the composite coating can be attributed to the resilient polymeric network formed by XDA and CS, as discussed in “FTIR” section, as well as the presence of HA/BG^69^. The developed composite coatings presented better adhesion than other reported coatings, that utilized biopolymers as well. M. A. U. Rehman et al.^62^ conducted the tape test (ASTM D3359-97 and B571-97) on composite coating of CHI/gelatin/Cu-doped-BG on a 316L SS substrate, the coatings were reported to delaminate at the edges. The better adhesion strength of the composite coatings is attributed to the defect-free coatings (microstructure), enhanced cross-linking capability of aldehyde groups in XDA, and etching of the substrate prior to the coatings which may have assisted in the mechanical and chemical linking of the composite coating with the substrate^43,70^. Thus, the coating was rated as “4B” according to the ASTM standard. Zhang et al.^71^ observed increased adhesion of coatings, coinciding with the uniform distribution of HA particles, of Bovine-HA deposited on Ti-12Cr substrate. Chen et al.^72^ utilized EPD to develop Alginate/BG coatings on 316L SS and remarked their adhesive strength to be “3B” according to ASTM D3359-B, after conducting the tape test.

#### Bend test

An ASTM B57197 bend test was used to assess the bending strength of the composite coating upon bending to 180°. Figure 4C, and D show the images of an optical microscope (Novex, Holland) captured after the bend test. The composite coating exhibited sufficient resistance to bending loads throughout substrates, as no delamination was observed even at the edges of the composite coating. In this study, composite coatings presented better adhesion strength upon bending compared to the^62^ where CHI-based composite coatings delaminated at the edges. The better adhesion strength of the composite coatings is attributed to the defect-free coatings (microstructure), enhanced cross-linking capability of aldehyde groups in XDA, and etching of the substrate prior to the coatings which may have assisted in the mechanical and chemical linking of the composite coating with the substrate^43,70^. Thus, the coating was rated as “4B” according to the ASTM standard.

#### Pencil hardness test

The adhesion strength of the XDA/CS/BG/HA/lawsone coating with the 316L SS was evaluated by the pencil test.

The findings showed the composite coating to be able to withstand a range of pencil hardness values without showing any signs of cracking or flaking till H. However, a slight powder delamination was observed when the coating was scratched with the 2H pencil, as shown in Fig. 4E. Said results are due to the presence of HA/BG^69^ and strong polymeric network of XDA and CS (discussed in “FTIR” section).

#### Scratch test

**Figure 5:**
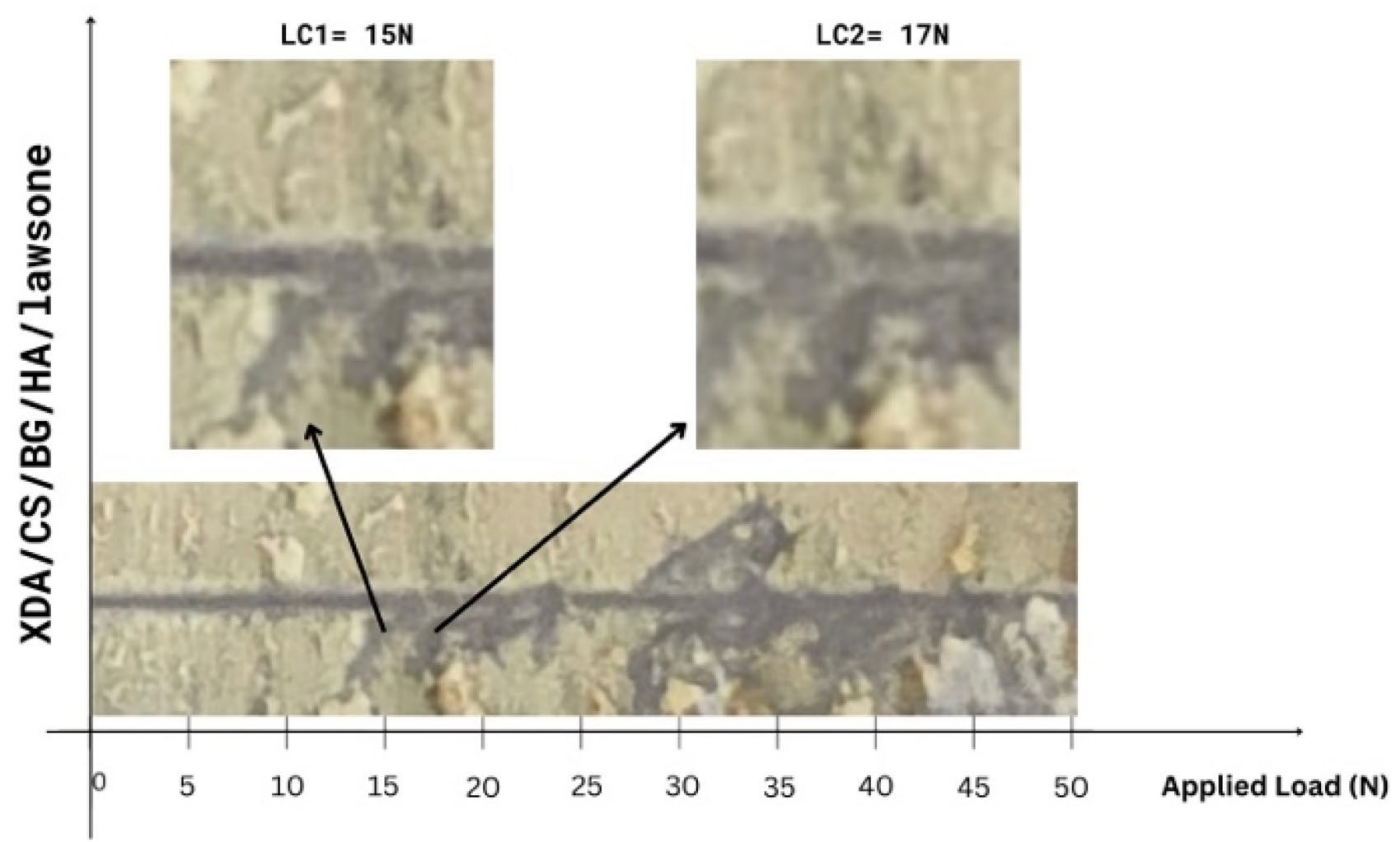
Results of the scratch test for XDA/CS/BG/HA/lawsone coating.

XDA/CS/BG/HA/lawsone composite coating adhesion strength was evaluated with the scratch test shown in Fig. 5. As shown in Fig. 5, XDA/CS/BG/HA/lawsone composite coating experienced a 15 N L_c1_ value, and circular cracks appeared on its surface, despite remaining intact. According to the measured value of second critical load (L_c2_), at L_c2_ there is still no significant delamination of coatings showing that the XDA/CS/BG/HA/lawsone composite coating has better adherence. After the L_c2_ composite coatings delaminated from the surface. A scratch test indicated that the composite coatings were suitable for designing coatings for orthopedic implant devices Fig. 5^73,74^.

### Surface properties

#### Wettability

**Figure 6:**
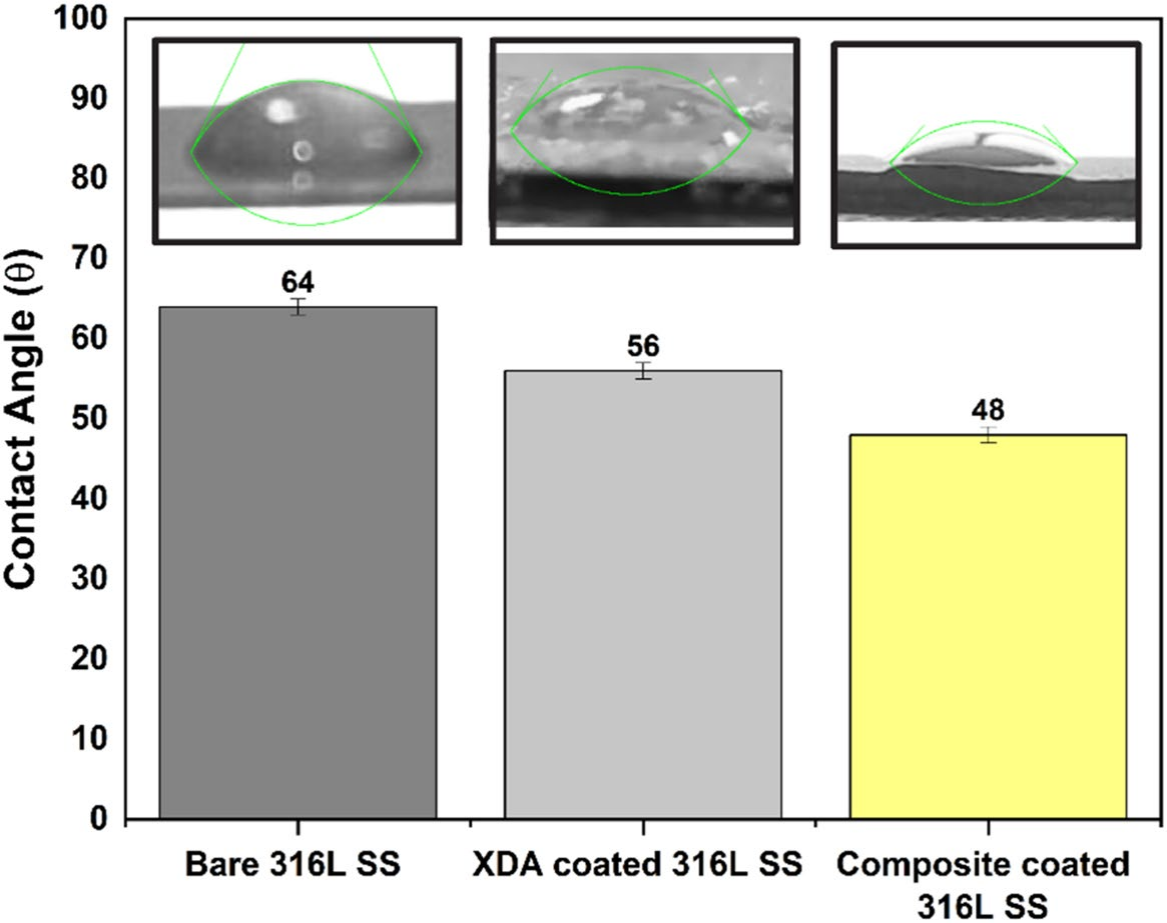
Contact angles comparison of bare 316L SS, XDA coated 316L SS, and XDA/CS/BG/HA/lawsone composite coating deposited on 316L SS (Digital images illustrating the presence of a water droplet on the bare surface of 316L SS, XDA coated 316L SS and XDA/CS/BG/HA/lawsone composite coating deposited on 316L SS).

The wettability of the surface was measured by contact angle, as shown in Fig. 6. The optimal contact angle for facilitating initial protein attachment typically falls within the range of (35°–80°)^75^. The measured values of bare 316L SS, XDA coated 316L SS and composite coating were 64° ± 1°, 56 ± 1° and 48° ± 4°, respectively. The contact angle of XDA coated 316L SS is lower than the bare 316L SS, indicating increased hydrophilic character introduced by the XDA coating. This enhanced hydrophilicity is attributed to XDA`s intrinsic properties, particularly its hydroxyl and aldehyde functional groups^76,77^.

Furthermore, the composite coating exhibited lowest contact angle signifies the highest-level hydrophilicity in comparison to XDA coated 316L SS and bare 316L SS Substrate, this could be due to the hydrophilic nature of HA, BG (silanol groups led to the hydrophilic nature of BG) and CS^78^. The presence of Sulfate and carboxylate functional groups in CS and hydroxyl groups in HA makes the composite coatings hydrophilic^10^. Additionally, XDA also showed a hydrophilic nature due to the introduction of aldehyde groups into the polymer chain, leading to enhanced hydrophilicity^77^. Chen et al.^72^ reported the relationship of contact angle and surface roughness, surface roughness increases as contact angle decrease and the reported results are in agreement with^72,79^. Manzur et al. shows the contact angle of Poly(lactic-co-glycolic) Acid/henna/Copper-doped Mesoporous bioactive glass nanoparticles (PLGA/henna/Cu-MBGNs) is 41° on Mg substrate which agrees with reported studies^57^. Shi et al.^79^ also conducted study of hydrophilicity on CHI/HA/GO coating on Ti substrate and reported a contact angle of 53.3 ± 1.3° and reported that enhanced hydrophilicity is desirable to facilitate the adhesion of cells and cell-adhesive proteins, promoting osteoconductivity.

#### Surface roughness test

Surface topography plays a significant role in influencing the cellular interaction with induced biomedical implants. Related cells and proteins should be able to adhere to the implant surface^49,80^. Surface topography was investigated by measuring the average surface roughness (Ra), maximum profile peak height (Rt), average maximum profile (Rz) of the XDA/CS/BG/HA/lawsone composite coatings deposited on 316L SS via EPD, as given in Table 2. The composite coatings exhibited relatively higher values of Ra, Rz, and Rt. The composite coatings exhibited the Rz values of 1.78 µm, which can allow the osteoblast cells to attach to the coated surface^81,82^ owe to the favorable surface topography. Osteoblast attachment and proliferation can be influenced by other factors than just surface topography. These factors include wettability and surface chemistry, which should also be considered. This study found that composite coatings had suitable wettability, roughness, and surface chemistry for protein attachment and later cell attachment and proliferation. To achieve optimal tissue integration, the growth and differentiation of the relevant cells are important (in the current case osteoblast cells)^83,84^.

**Table 2:** Surface roughness parameters (Ra, Rt, and Rz) of XDA/CS/BG/HA/lawsone composite coatings on bare 316L SS and bare 316L SS.

Sample	Average roughness (Ra) μm	Maximum profile peak height (Rt) μm	Average maximum profile (R_z_) μm
Bare 316L SS	0.18 ± 0.009	2.67 ± 0.04	1.23 ± 0.17
Composite coated 316L SS	0.32 ± 0.015	2.94 ± 0.27	1.78±0.13

### Chemical properties

#### EDX analysis

**Figure 7:**
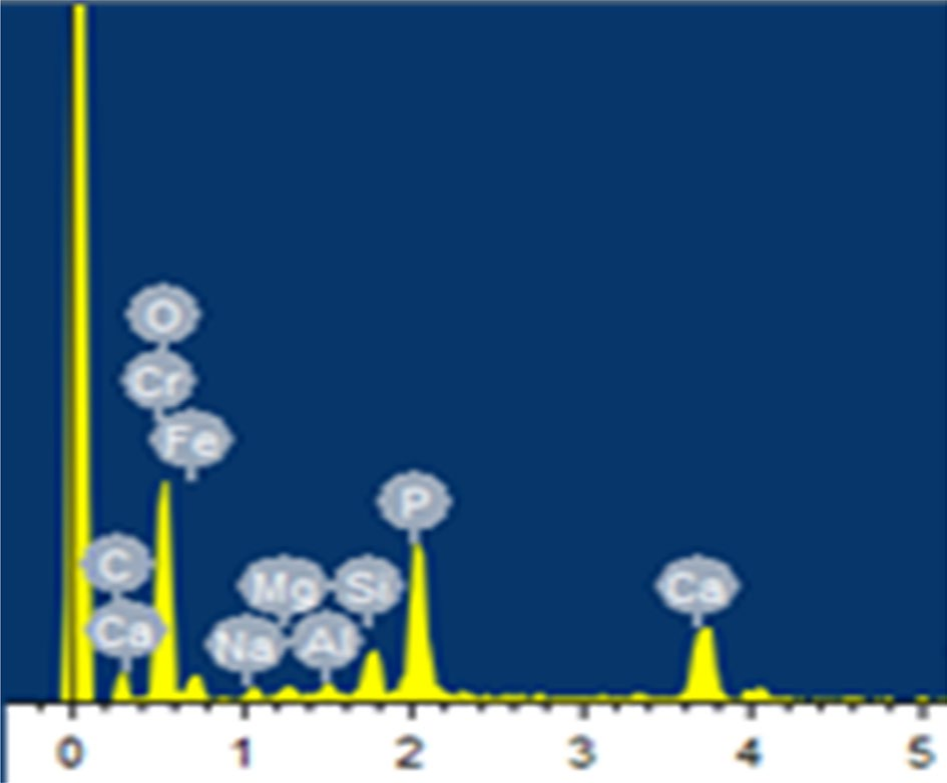
EDX spectrum of XDA/CS/BG/HA/lawsone composite coating deposited on 316L SS.

EDX analysis indicated the presence elements attributed to the biopolymers (XDA/CS), bioceramics (HA/BG) and lawsone. The peaks of O and Carbon (C) may denote the subsistence of CS and XDA in the composite coatings, as shown in Fig. 7. Although, it is not accurate to spot the C and O via EDX. Therefore, to verify the subsistence of XDA and CS in the composite coatings, FTIR was executed. Additionally, the occurrence of HA in the coating was verified by the identification of peak spots, associated with P and Ca. Furthermore, the area EDX analysis portrayed Mg, Si and P indicating the presence of BG in the composite coatings. Although, the Ca and P are present in HA and BG. The appearance of Si is associated with the presence of BG in composite coating.

#### FTIR analysis of composite coatings

The identification of the aldehyde groups in XDA can be confirmed by FTIR spectroscopy. The region of 1730–1750 cm^−1^ shows an evident peak which identify the aldehyde group in XDA^70^. In addition, the peak in the region of 1600–1650 cm^−1^ associated to the C=C stretching vibration and peak at 2978 cm^−1^ is associated with C–H stretching vibrations, respectively^10^. Moreover, the band of O–H stretching vibrations appeared in the range of 3200–3600 cm^−1^. The peaks in the region of 1050–1100 cm^−1^ correspond to C–O–C stretching vibrations^10^.

**Figure 8:**
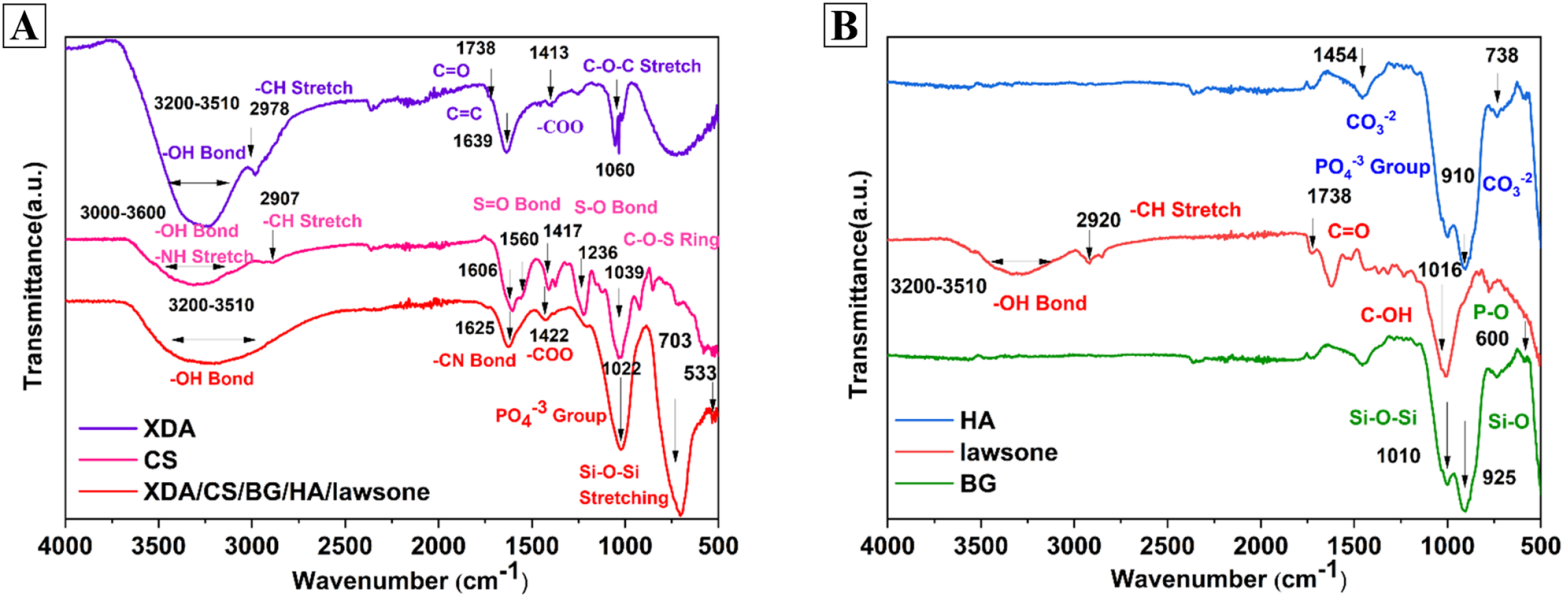
FTIR spectra of: (**A**) XDA, CS and XDA/CS/BG/HA/lawsone and (**B**) HA, lawsone and BG.

In FTIR spectra of CS, the peaks in between 3000–3600 cm^−1^ represent –NH and –OH stretching vibrations. The broad peak appeared at 2907 cm^−1^ correspond to the C–H bond^13^. The sulfate region of CS can typically be found between 1000 and 1300 cm^−1^ as shown in Fig. 8A. This region indicated the presence of a strong peak at 1236 cm^−1^, attributed to the disproportioned and uneven stretching vibrations of the sulfate groups (S–O bond))^85^. The peak at ~ 1039 cm^−1^ can also be observed, which corresponded to the symmetric stretching vibration of the sulfate groups (C–O–S ring))^85^. The amide region of CS was found between 1700 and 1500 cm^−1^. The peak at 1606 cm^−1^ introduced due to the amide I vibration of the peptide backbone^86^, similarly peak at around 1560 cm^−1^ was introduced due to the amide II vibration of the peptide backbone^85,87^. FTIR spectra of bioactive glasses showed Si–O–Si stretching vibration, at ∼703 cm^−1^and 468 cm^−1^ and Si–O stretching of nonbridging oxygen atom at ∼925 cm^−1^^88,89^. This peak is manifesting the SiO_2_ network structure in BG, which is critical for its bioactivity. The phosphate peak (PO_4_^−3^) appeared at ∼610 cm^−1^^89^.

The peak at ~ 910 cm^−1^ in HA, is accredited to the stretching vibration of phosphate (PO_4_^−3^) groups in HA. An extensive band in the range of 500–600 cm^−1^, depicted bending vibration of phosphate (PO^−3^) groups in HA^90^. A weak peak at around 738 cm^−1^, corresponded to the deformation vibration of carbonate (CO_3_^−2^) groups in HA as shown in Fig. 8B. Moreover, a solid peak for carbonate (CO_3_^−2^) appeared at 1454 cm^−1^^90,91^. The infrared spectra of lawsone with transmittance peaks showed at ∼3320 cm^−1^ indicating an O − H bond^92^. The peak spot at about 1755 cm^−1^ denotes the carbonyl group (C=O), while the aliphatic C–H stretching peaks associated with ∼2925 cm^−1^. The phenolic groups in henna were evident by the climax at ∼1000 cm^−1^^93^.

The FTIR spectra of composite coating showed the genesis of Schiff base between XDA and CS^86^. XDA exhibited characteristic peaks of the aldehyde group (C=O) at around 1730–1740 cm^−1^. However, the amine group (N–H) in CS appeared at around 3300–3400 cm^−1^. After the crosslinking reaction, a new peak corresponding to the imine group (–C=N–) in the Schiff base appears at a typical range of 1620–1660 cm^−1^^94^ This shift in the peak position from the aldehyde and amine groups to the imine group can serve as an indicator for the formation of the Schiff base between XDA and CS. The -OH band existed in the final composite coating showing the presence of henna and XDA. The overlapping peaks at 1022 cm^-1^ is attributed to phosphate group of HA and phenolic group of henna^90,93^. However, the peak of dispersed BG in the composite coating appeared at 703 cm^−1^ corresponded to Si–O–Si stretching vibrations^95^. The phosphate group peak due to HA appeared at 533 cm^−1^^86^.

### Corrosion behavior

#### Potentiodynamic polarization

**Figure 9:**
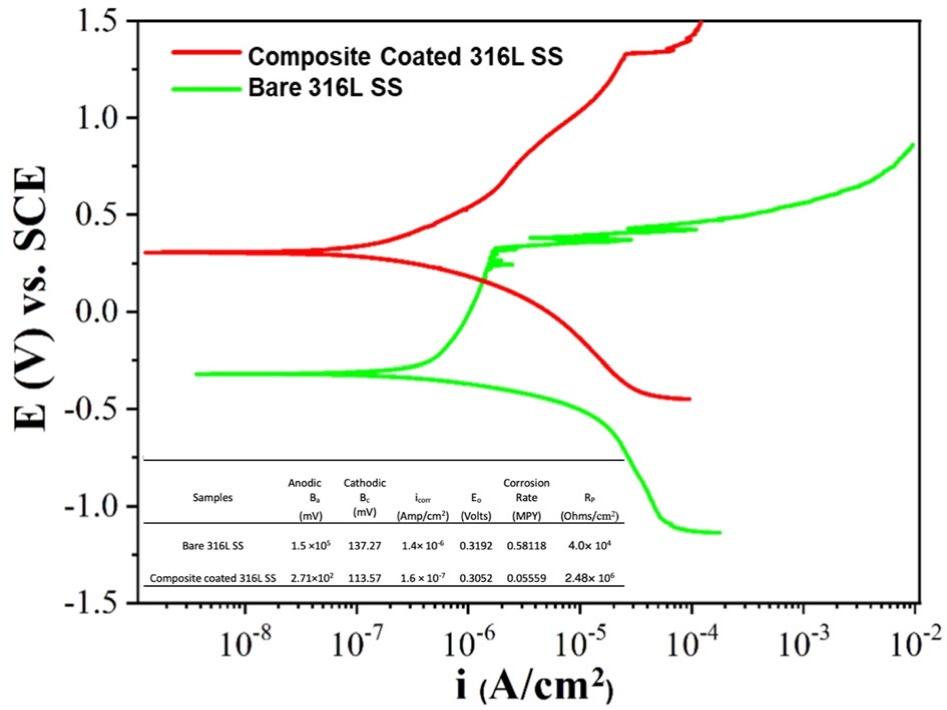
Electrochemical polarization of XDA/CS/BG/HA/lawsone and bare 316LSS.

The potentiodynamic polarization behavior of bare and XDA/CS/BG/HA/lawsone coated 316L SS in SBF is represented in Fig. 9. The values of E_corr_ and i_corr_ were determined using the Tafel method. From Fig. 9 the composite coated 316L SS shows the cathodic slope is lower as compared to the bare 316 L SS. Similarly, the anodic slope of the composite coated 316L SS is suppressed by three orders of the magnitude and have a good agreement with the cathodic slope. The composite coated 316L SS shows reduced corrosion current density (i_Corr_) and higher corrosion potential (E_corr_) than bare 316L SS. The polarization resistance (R_p_) of the XDA/CS/BG/HA/lawsone coated 316L is increased in two ordered of the magnitude than bare 316L SS. This indicates that the composite coating provides the significant corrosion resistance to 316L SS in SBF solution^13,96,97^.

#### Electrochemical impedance spectroscopy (EIS)

**Figure 10:**
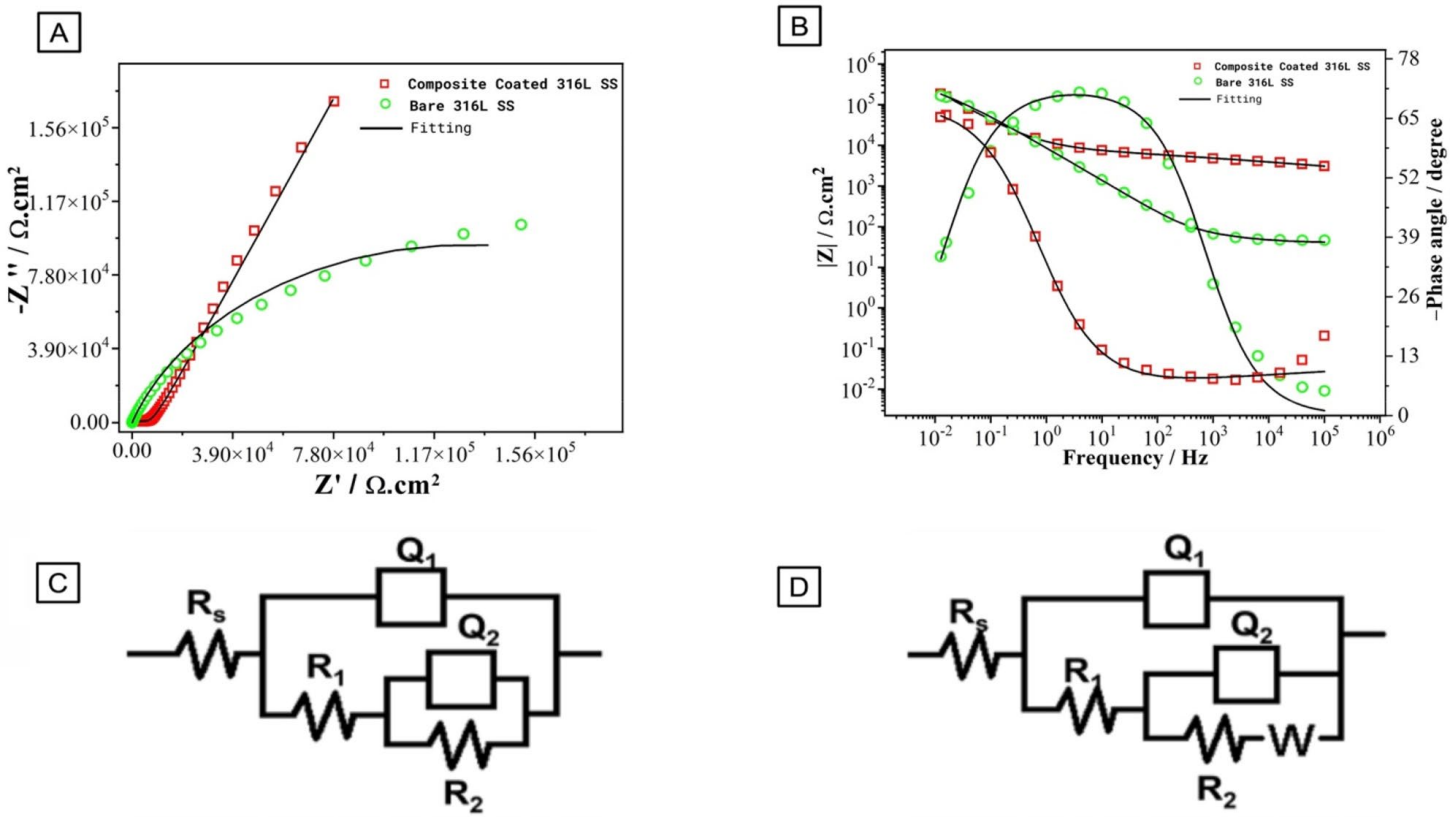
(**A**) Nyquist plot of XDA/CS/BG/HA/lawsone and bare 316L SS, (**B**) Bode plots of XDA/CS/BG/HA/lawsone and Bare 316L SS, (**C**) Equivalent electric circuit of bare 316L SS and (**D**) Equivalent electric circuit of XDA/CS/BG/HA/lawsone.

The plot of the EIS of uncoated 316L SS compared to XDA/CS/BG/HA/lawsone coated 316L SS is shown in Fig. 10A. The Nyquist plots of the bare and coated samples present a high semi-capacitive arc. It indicates that the same process of corrosion is occuring at a lower rate for the coated substrate^47^. Figure 10B shows the impedance values are almost equal at low frequencies (úZê_0.01 Hz_), but at higher frequency, coated 316L SS has higher impedance values—dispensing information about the electrode/solution interface. Bode phase plot shows coated 316L SS exhibits an increased capacitive response over the broad frequency range with a lesser phase angle than bare 316L SS^59,98^.

Figure 10C, D shows that the electric equivalent circuits (EEC) Rs(Q_1_(R_1_(Q_2_R_2_))) and R_s_(Q_1_(R_1_(Q_2_(R_2_W))) indicates the electrochemical activity between uncoated and coated 316L SS. R_s_(Q_1_(R_1_(Q_2_(R_2_W)))) is the preferred choice for coated 316L SS. According to the reported data, numerous interpretations have been provided for the circuit components^99^. Specifically, Q_1_ and R_1_ represent the porous layer capacitance and resistance, while Q_2_ and R_2_ show the barrier layer resistance when connected (series) with a Warburg diffusion element (W). The term R_s_ represents solution resistance. As shown in Fig. 10A, certain diffusin in Nyquist plot introduced the Warburg diffusion component.

Table 3 shows that the R_1_ porous layer has a higher corrosion resistance than R_2_, thus BG/HA/lawsone contributed more to corrosion resistance than R_2_ resistance. Potucek et al.^100^ indicated the solution chosen has significant impact on the resistance R values generated from the EIS data, while the capacitance (C) values can be used to accurately determine the electrochemical properties associated with the solution selected, irrespective of the solution condition. Equation 1 allows us to calculate the different elements`s equivalent capacitance values in Fig. 10C, D by combining values (Q and n) with their respective resistances.

**Table 3:** EIS circuit elements fitted from EIS data in SBF.

Samples	R_**s**_	Q_**1**_	n_**1**_	R_**1**_	Q_**2**_	n_**2**_	R_**2**_	W
	(Ω cm^2^)	(µΩ^−1^s^n^ cm^2^)		(kΩcm^2^)	(µΩ^−1^s^n^ cm^2^)		(kΩcm^2^)	(Ωs^−1/2^)
Bare 316L SS	40.28	1.9	0.75	58.3	4.6	0.9	303	–
Composite coated 316L SS	2751	1	0.4	4310	0.66	0.7	380	1.16 × 10^3^

1 $$C={Q}^{1/n}{R}^{(1-n)/n}$$

### Biological studies

#### In-vitro SBF studies

**Figure 11:**
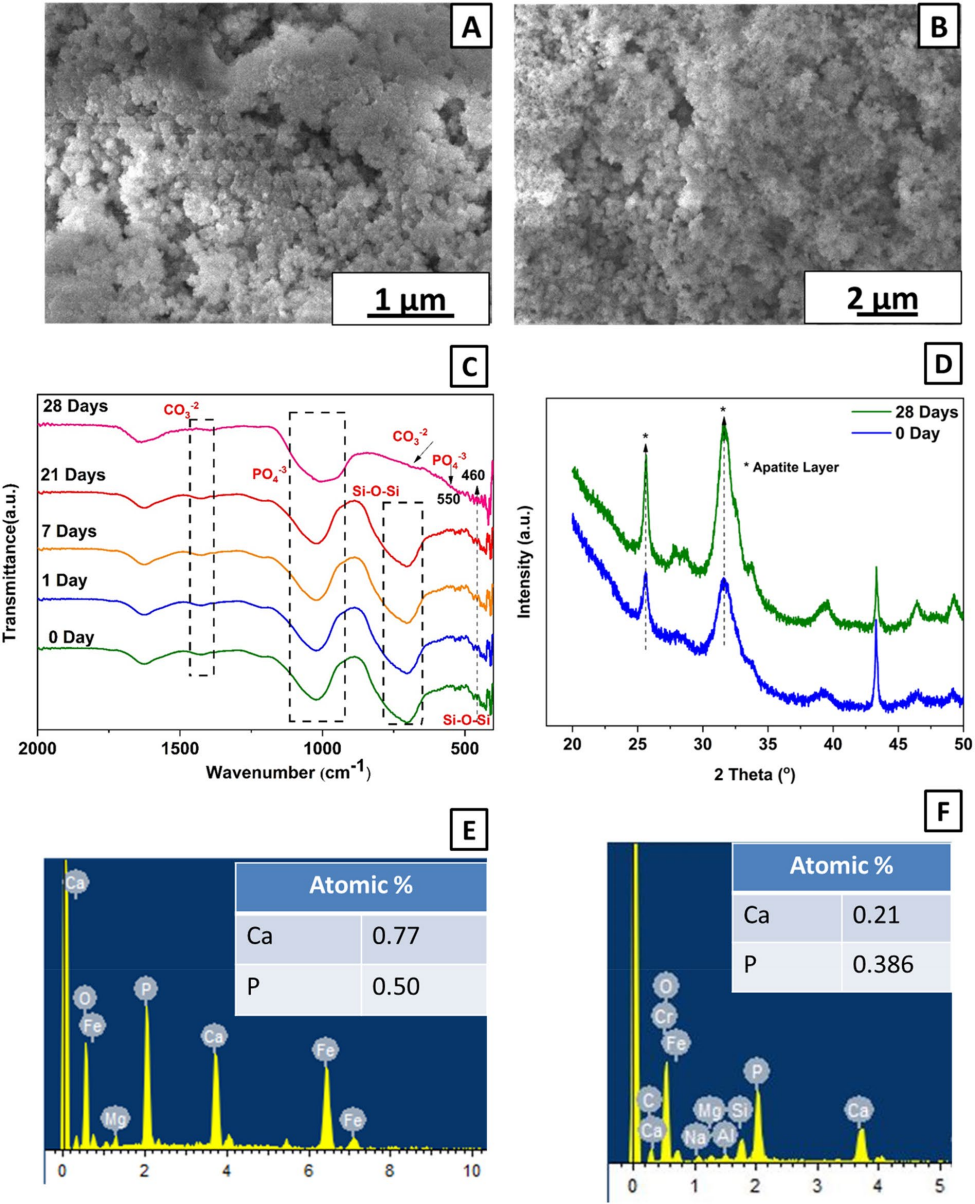
(**A**) SEM image of composite coatings in SBF for 28 days at 25kX, (**B**) SEM image of composite coatings in SBF for 28 days at 10kX, (**C**) FTIR spectra before and after immersion in SBF for 1, 7, 21 and, 28 Days and, (**D**) XRD pattern for 0 Day and 28 Day, (**E**) EDX analysis of composite coating immersed in SBF for 28 days with Ca/P ratio of 1.54 and (**F**) EDX analysis of composite coating before immersion in SBF with Ca/P ratio of 0.544.

The XDA/CS/BG/HA/lawsone composite coating was immersed in SBF to track the possible changes in the morphology and the formation of the apatite-like layer on the surface of the coatings. It is widely accepted that the genesis of apatite-like layer indicates the osteoinduction and osteointegration of the designed implants^84^. The HA layer formation was confirmed via FTIR, SEM, EDX and X-ray diffraction (XRD). Dissolution of the BG particles results in the release and diffusion of Ca and P ions, the ionic exchange that then happens assists in the formation of apatite-like structure on the surface of the coatings (Fig. 11A, B)^20,21,101^. The dissolution of BG was evident by the decrease of the intensity of silica peaks in the FTIR spectra (the reduction in silica peak may also be credited to the initiation of the HA layer after being dipped in SBF). The magnitude of the silica peaks reduces with the increase in immersion time whereas the intensity of phosphate bands increases (Fig. 11C). After 28 days of immersion in SBF, the FTIR spectra faded away BG-associated Si–O–Si elongated peaks^102^. The bioactive glass peaks were formed at 460 and 703 cm^−1^ in pure composite coating and after immersion in SBF till 21 days, as shown in (Fig. 11C). The carbonate (CO_3_^−2^) and phosphate (PO_4_^−3^) peaks attributed to HA formation in SBF were observed after 28 days. The slightly shifted and intensified carbonate peaks appeared at 680 and 1415 cm^−1^. Moreover, broad, and more intense peaks of phosphate group appeared at 550 and 980 cm^−1^. Agathopoulos et al.^102^ also reported the appearance of HA layer associated peaks in FTIR after immersion in SBF. The reported results are in agreement with^103,104^.

The possible mechanism for the formation of HA crystals upon dipping in SBF is attributed to the release of Ca and P ions from the composite coatings, which then react with the SBF ultimately devising a concentrated solution of Ca Phosphate. Thus, resulting in the formation of HA crystals on the surface of the coating through nucleation and growth^105^. The HA layer formation on composite coating after immersion in SBF for 28 days was first validated by FTIR followed by SEM analysis.

HA layer formation on BG coatings was also confirmed using FESEM after immersion in SBF, as shown in (Fig. 11A, B). The XDA/CS/BG/HA/lawsone coatings are designed to create a bioactive layer on implant surfaces (to induce mineralization), which is crucial for successful osteointegration^106^. FESEM analysis showed the appearance of typical spherical or needle-shaped morphology.

XRD was utilized to examine XDA/CS/BG/HA/lawsone coatings immersed in SBF for 28 days to verify the existence of a HA layer, as depicted in Fig. 11D. The results of the XRD analysis unequivocally substantiated the formation of a HA layer before immersion in SBF due to the presence of HA in the developed coatings. The increase in the intensities of HA associated peaks after immersion in SBF for 28 days confirmed the formation of HA layer due to BG in coating^107^. The XRD pattern exhibited a peak corresponding to HA at angles of 25.8° and 31.5°^108,109^.

EDX analysis after 28 days of immersion in SBF showed the peaks of Ca and P while Si peak vanished (agrees with FTIR analysis). The Ca/P ratio calculated from the EDX analysis before and after immersion in SBF was 0.544 and 1.54, respectively as shown in Fig. 11E, F. Ca/P ratio of 1.54 after immersion in SBF is very close to the stoichiometric ratio of the HA^101^. The results agree with^110,111^

#### Antibacterial studies

**Figure 12:**
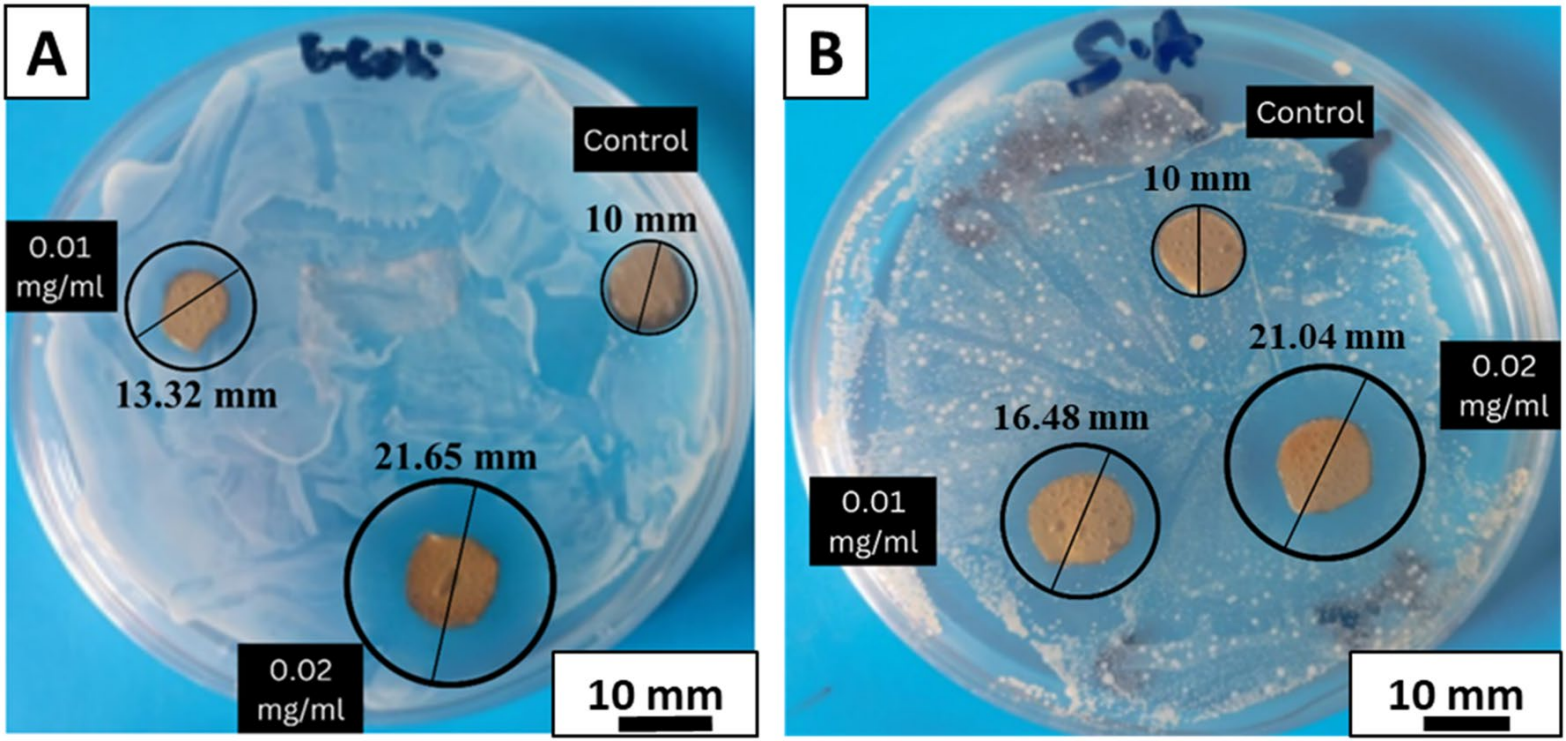
Inhibition halo tests for the XDA/CS/BG/HA/lawsone and XDA/CS/BG/HA (control) coatings with (**A**) *E. coli* and (**B**) *S. aureus*.

Significant antimicrobial effects of composite coatings were seen because of lawsone and naphthoquinone release. Figure 12A shows that XDA/CS/BG/HA coated 316L SS did not form any zone of inhibition, while 0.01 mg/mL and 0.02 mg/mL lawsone loaded composite coated samples showed the zones of 13.32 mm ± 0.5 mm and 21.65 mm ± 0.5 mm against *E. coli*, respectively. While Fig. 12B shows that XDA/CS/BG/HA 316L SS couldn’t show any bacteriostatic or bactericidal properties while 0.01 mg/mL and 0.02 mg/mL lawsone loaded composite coated samples exhibited zones of 16.48 mm ± 0.5 mm and 21.04 mm ± 0.5 mm against *S. aureus*, respectively. The clear zone of inhibition portrayed that 0.02 mg/mL of lawsone was enough to create strong antibacterial effect. Lawsone (2-hydroxy-1,4-naphthoquinone) the major component of henna, generates reactive oxygen species (ROS) and shows to apoptotic cell death of bacteria^112^. The results are consistent with those reported by Rubiay et al.^113^.

**Figure 13:**
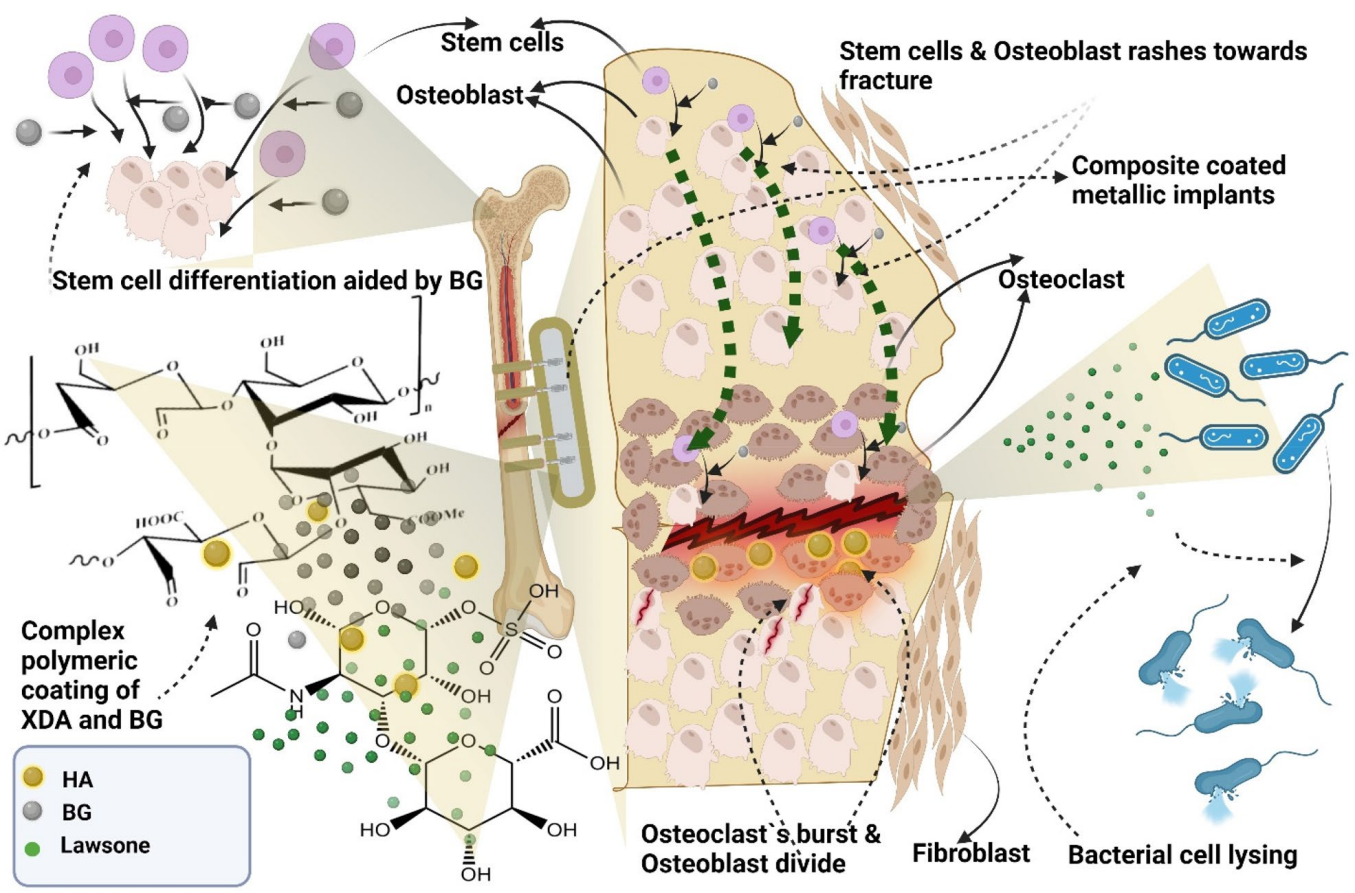
The proposed mechanism of biochemical action of XDA/CS/BG/HA/lawsone composite coatings to facilitate bone regeneration and to inhibit the growth of biofilm formation.

Novel and functional bio-composite coating development has been a success. Apatite induction was confirmed through *in-vitro* SBF studies, while antibacterial potential due to the release of lawsone was established and evaluated through Kirby–Bauer test against *E. coli* and *S. aureus*. Corrosion resistance of 316L SS was enhanced by first order of magnitude, examined via Potentiodynamic polarization scan and EIS analysis. The composite coatings developed in the current study showed the strong potential to coat orthopedic implant devices. The composite coatings can provide bone regeneration and can prevent biofilm formations. The future studies will involve cell biology, biomarkers quantification, alkaline phosphate activity, drug release kinetics, quantitative microbiology studies and in-vivo studies. It is expected that the designed coatings will help in the differentiation and proliferation of the osteoblast cells which will eventually help in the growth of bone tissues. The biochemical process aided through XDA/CS/BG/HA lawsone is proposed to overcome the challenges associated with orthopedic implants, as shown in Fig. 13.

## Conclusions

The XDA/CS crosslinking in the polymeric network was confirmed by FTIR analysis, resulting in the formation of a robust coating of XDA/CS/BG/HA/lawsone (coating thickness ~ 3 µm) using EPD. The results of the pencil hardness test, tape test, scratch test, and bend test confirmed suitable adhesion strength of the composite coating with the 316L SS substrate. The bioactivity and antibacterial tests, combined with the potentiodynamic polarization assessment, demonstrated that the 316L SS coated with XDA/CS/BG/HA/lawsone exhibited promising characteristics for the development of orthopedic implants. The coated substrate showed significant corrosion resistance, as well as apatite induction and antibacterial potential, making it a highly attractive prospect for further investigations and eventually the translation to the clinic. Overall, the results suggested that the fabricated coated implant has the potential to be considered to design orthopedic implant devices (Table 4).

**Table 4:** Comparative analysis of reported results with previously reported results.

Experiment	Results	Literature	Summary
Adhesion tests	4B adhesion strength confirmed via tape and bend test. Adhesion suitable for orthopedic implants	Cheng et al.^72^—Alginate/BG coatings, ‘3B’ Ur Rehman et al.^62^—CHI/gelatin/Cu-doped-BG coatings, ‘4B’	Increased adhesion due to strong polymeric network of XDA/CS and uniform particle distribution
Contact angle	Substrate and coating contact angles respectively; 64° ± 1° and 48° ± 4°	Shi et al.^81^—CHI/HA/GO coating, 63.3° ± 1.3°	Lower values of contact angle due to hydrophilic nature of constituents
Surface roughness	Coating demonstrated higher surface roughness than bare substrate Ra (coating) = 0.32 ± 0.015 µm Ra (bare) = 0.32 ± 0.015 µm	Cheng et al.^72^—Alginate/Bioglass coatings Ra (coating) = 0.89 ± 0.030 µm Ra (bare) = 0.63 µm	Surface roughness promotes osteoblast cell attachment and proliferation
Corrosion behavior	Lowered corrosion current density than bare substrate iCorr (coating) = 1.6 × 10^−7^ iCorr (bare) = 1.4 × 10^−6^	Khosravi et al.^114^—GO/PCL-gelatin-forsterite 3% iCorr (coating) = 0.017 × 10^−6^ iCorr(bare) = 0.59 × 10^–6^	Increased corrosion resistance of implant
Antibacterial	Clear inhibition zones against *E. coli* and *S. aureus*	Batool et al.^58^—Zein/Zn-Mn-doped-MBGNs Complete inhibition against *S. aureus,* partial inhibition against *E. coli*	High antibacterial efficacy
In-Vitro bioactivity	Apatite layer development after 28 days	Farrokhi-Rad et. al^115^—HA/Titania-nanocomposite coating on Ti Apatite layer development after 7 days	Slower bioactivity

## Data Availability

The datasets used and analyzed during the current study are available from the corresponding author upon reasonable request.
